# Biomarkers in Child Mental Health: a bio-psycho-social perspective is needed

**DOI:** 10.1186/s12993-015-0076-6

**Published:** 2015-09-30

**Authors:** Aribert Rothenberger, Luis Augusto Rhode, Lillian Geza Rothenberger

**Affiliations:** Child and Adolescent Psychiatry, University Medical Center Goettingen, von-Siebold-Str. 5, 37075 Goettingen, Germany; Kinder- und Jugendpsychiatrischer Dienst Thurgau/Schweiz, Bahnhofplatz 69b, 8500 Frauenfeld, Switzerland; Child and Adolescent Psychiatric Division, Hospital de Clínicas de Porto Alegre, Federal University of Rio Grande do Sul, Porto Alegre, Brazil; Institute for Developmental Psychiatry for Children and Adolescents (INCT-CNPq), Porto Alegre, Brazil

## Rethinking the past

There is still an increasing interest in biomarkers in (child) psychiatry although some disappointment took place within the last decades for those who expected simplistic solutions. For example, the Dexamethasone Suppression Test (DST) showed initial promise to diagnose endogeneous depression, drug response and clinical relapse, but finally it was of limited clinical value and abandoned [1]. The same holds true for former expectations to find a certain gene for a certain mental health disorder and treat it by genetic therapy. Fortunately, neurobiological research in psychiatry has overcome such simplistic models. Meanwhile our scientific knowledge has increased tremendously and we discuss the highly complex pathophysiological background of mental disorders on a broad empirical basis. Hence, to find biomarkers of clinical utility we should rethink our approach. Not only a fresh and critical look at the biological underpinnings of mental disorders is recommended, but also one should take into account aspects of their interaction with environment and translation into patient care.

There are many papers just comparing people with typical development with a group of psychiatric patients to report an association between one parameter or several single biological parameters (e.g. genes, brain oscillations, cortisol, oxytocin, BDNF, event-related potentials, fMRI regions) and the disorder. They frequently conclude that the investigated parameter “may be a promising biomarker” [2, 3, 4]. This approach is more or less on the wrong track, if no further steps for testing the validity of the biomarkers are undertaken, as is rarely the case. Especially, things become more challenging when developmental aspects have to be added in studies with minors.

In order to understand why earlier research paved such a simplistic way one should go back to the point where and how the idea of biomarkers in psychiatry was promoted. Since decades psychiatrists are trying to elucidate the biological underpinnings and neuroscientific dynamics of mental disorders in children (e.g. [5, 6]). This approach is still valid and should be followed further in parallel to the research on the objectives related to biomarkers. With the advent of new technologies like brain mapping, neuroimaging, genetics, epigenetics, proteomics, neurotransmitters, metabolic assessment, neuropsychology, brain-computer-interface, many researchers and clinicians expressed the hope to advance the field by developing a biologically guided psychiatric classification, diagnostic system and treatment recommendation [1, 7]. In other words, a paradigm shift in psychiatry with a promise of rescuing the validity of symptom based psychiatric diagnosis through its link to the pathophysiology of mental disorders. However, so far, the limited robust facts did not allow for implementing such an approach into DSM-V.

Moreover, Peterson [8] stated that the NIMH promoted Research Domain Criteria (RDoC), i.e. a mechanistically and neurobiologically oriented new approach to psychiatric nosology, is still “premature, … theoretically problematic and its measures psychometrically untested”. Thus “it seems unlikely that a single RDoC domain validly represents a single link in the causal pathway from genes to behavior.” Hence, it can hardly be expected that related biomarkers will be clinically more relevant than those related to DSM-V categories and the intrinsic dimensions.

## Developing a guideline for new research

Nevertheless, the Biomarkers Definitions Working Group [9] circumscribed what we should be looking for in the future, namely “A characteristic that is objectively measured and evaluated as an indicator of normal biological processes, pathogenic processes or pharmacologic responses to a therapeutic intervention”. A biomarker can be used as a diagnostic tool, a tool for staging a disease, an indicator of disease prognosis, a substitute for a clinical endpoint or for prediction and monitoring of clinical response to an intervention. That means, a biomarker may either represent characteristics of a trait or a state parameter. Unfortunately, this defining group addressed insufficiently what should be the adequate psychometric performance to be fulfilled by these biomarkers. A psychometric profile with high values for sensitivity, specificity, reproducibility, negative/positive predictive value, discriminant validity, effect sizes should be demanded; see also (Thome et al. [10]). These are essential preconditions in order to estimate clinical utility. Another essential for clinical decisions is treatment relevant measurement of psychosocial impairment which cannot be delivered by biomarkers but by clinical interview only, although it seems plausible that social behavior and stress are reflected in some biological parameters like cortisol, prolactin, heart-rate variability, pupillary reactivity, skin conductance level and serotonin transporter gene [11]. Are these challenges the reason why it is still so difficult for biological psychiatry to develop valid clinical tests and why we hear from authors only statements like the “Zeitgeist” formulation that a certain biological measurement showed some association with a disorder or process and thus “may be a promising biomarker”—while for further validation nothing happens afterwards? Specifically, this holds true when, for example, methodological hurdles are high in minors where brain development, symptom heterogeneity with its changes along the lifetime and family factors modulate the results.

Kapur et al. [1] suggest a new approach: “Rather than seek biomedical tests that can ‘diagnose’ DSM-defined disorders, the field should focus on identifying biologically homogenous subtypes that cut across phenotypic diagnosis—thereby sidestepping the issue of a gold standard. To ensure clinical relevance and applicability, the field needs to focus on clinically meaningful differences between relevant clinical populations, rather than hypothesis-rejection versus normal controls” (p. 1, key-words: discriminant validity, differential diagnosis) and “supplement, rather than replace, the symptom-driven diagnosis” (p. 3; key-words: hubs of psychopathological profile). Concerning biomarkers, the authors think merely of a dimensional diagnostic and treatment approach across disorders as it is already done in pragmatic daily clinical practice, i.e. taxonomists and biomarker researchers could learn from experienced clinicians in order to develop new hypotheses and design their studies accordingly. Or, as Rommelse and de Zeeuw [12] stated: “… neurobiological investigations can help us gain a better understanding of the biological vulnerabilities that may underlie ADHD in a specific patient or that may moderate the response to treatment thereby contributing to better and more effective treatment”. For example, in children with ADHD the contingent negative variation (CNV), which is an electrical brain activity associated with cognitive preparation, when measured before neurofeedback (NF) training explained about 30 % of the variance related to the reduction of ADHD symptoms by NF [13]. This might be a first step in order to develop personalized treatment guiding biomarkers.

Meanwhile, clinicians and researchers have realized that the biology behind clinical entities, even if these are symptomatically homogeneous, is quite complex. Hence, they are looking for “complex biomarkers” like multiparametric proteomic disease signatures in order to find tools for diagnostics or prognosis of risks [14].

For example, (1) the project PRONIA (Personalized Prognostic Tools for Early Psychosis Management; http://www.pronia.en) plans to establish a computerized self-learning algorithm which combines data from neuropsychology, brain imaging, genetics and clinical interviews [15]. Similarly, others work on learning (vector) machine approaches and feature reduction techniques in neuroimaging to generate clinical useful biomarkers [16, 17] or try to find “more accurate imaging-based classifiers for neuropsychiatric disorders” which e.g. may identify “neural pathways with aberrant morphological features associated with Tourette Syndrome in children and adults” [18, 19]. Also, the validity of the RDoC domains during child development (as well as DSM/ICD psychopathological clusters/profiles) need further investigations in order to better verify on which basis the biomarker in question is grounded and tested. Therefore, a better and more detailed knowledge on cortico-subcortical circuits in children is demanded. “These neuronal preconditions (necessary but not sufficient) play an important role regarding the understanding and treatment of behavioral problems in children” [20]. The authors could show by functional imaging that “the sensorimotor and associative circuit may be discriminated by their laterality and rostrality characteristics already in healthy minors”; probably a helpful step towards a better tuning for brain-behavior relationship within the framework of clinical assessment and thus for biomarker research. However, all this technical progress has not yet reached the level for translation into clinical use.

Further, (2) a “polygenic liability score” across mental disorders might help to find the magnitude of overlap, supporting the shift from categorical to merely dimensional approaches/constructs as outlined in the Research Domain Criteria (RDoC) initiative of the National Institute of Mental Health (USA) (see [21]). Although there exist some grounds for optimism, the translational science of child psychiatric genetics may be currently no more than a distant dream [22]. There have many methodological questions to be solved in order to find the most powerful composition of biological markers for a certain goal. For example, Hebebrand and Antel [23] ask, if it would make sense to analyze a biomarker like BDNF, if GWAS data for the respective gene are negative? In this context another field for biomarker research might be of interest.

*Endophenotypes*, which are way stations on the pathway between genes and behavior, and can measure objectively (e.g. via neurophysiology, endocrinology, biochemistry etc.) the biological processes related to emotions, cognition and behavior. Endophenotypes could also help to identify people at risk for psychopathology (e.g. non-syndromale siblings of ADHD patients) and thus be used for prevention [21]. In child psychiatry preliminary data in ADHD is available [24], awaits replication and psychometric evaluation. Currently no biomarkers for ADHD have achieved the status of clinical utility as a diagnostic tool [4, 10] but there exists a FDA-clearance since 2014 for the Qb-Test (Quantified Behavior Test). The latter is a motion-tracking device and meant to provide a more objective dimensional measure of attention and hyperactive problems than clinical observation and rating scales and thus be “an aid in the evaluation of treatment interventions in people with ADHD” and hopefully avoid mislabeling of children [25]. At the time being, the evidence base is still too weak to draw firm conclusions about its clinical added value.

Similar, a commercial blood test (VeryPsych™) the “.. first validated biological blood test for diagnosis of schizophrenia … ” [26] also needs to present a more robust evidence base before further clinical use should be discussed. The same holds true for EEG theta/beta ratio of the Neuropsychiatric EEG-Based Assessment Aid (NEBA) System which got the FDA permit to marketing the “first brain wave test to help assess children and teens for ADHD” (FDA News Release, July 15, 2013). Fortunately, the NEBA-System is recommended to be used along with other clinical information only!

## Beyond biology

The latter three examples (Qb-TEST, VeryPsych™, NEBA) clearly show, that the discussion around biomarkers needs to be extended beyond scientific rigor of the chosen biological parameters, since with the involvement of commercial companies aspects of marketing, trading, profit orientation and public health politics come into play and may speed up translational processes with the risk that these go awry when differences between common sense, commercial sense, political sense and science develop [27]. In this respect, a key issue has to be solved, namely, when is the scientific evidence for a biomarker sufficiently solid for translation into patient benefits, and if so at which point in time should be decided to act or not to act? “There are penalties for both, acting too soon and not acting soon enough” [27]. With respect to biomarkers, such a decision will not only be guided by the highest quality of research with best evidence, but the interpretation and translation of the scientific results is also influenced by clinical, psycho-social and political factors; the more, the younger people are; specifically, mental health of minors needs the shelter of family and society in order to avoid shaping of this translational process to the disadvantage of children. It follows, that ethical dilemmas may raise, which need to be analyzed. “And the analyses should not be viewed as merely the ‘social work’ adjunct to the ‘hard science’. Research into the social and ethical processes of translation, and into the challenges that are often faced, should inform the work of researchers themselves and can help to ensure that this research does result in improvements in social practice” (Singh and Rose [28], p 203). Hence, the authors raise three main concerns about the potential use of psychiatric biomarkers:

1. What is the best way to communicate the idea of “risk profile”, and how might this affect personal identity?
2. How can the complexity of mental health problems be retained when using information about biomarkers in the clinic and community?
3. What issues might arise from the commercialization of biomarkers, and how should they be addressed?

We would like to add a fourth comment (see also above):

4. How can the process of biomarker translation into practice be guided/regulated to be scientifically sound and psycho-socially adequate?

In their paper, Singh and Rose discuss the points 1–3 in depth, always side by side with the well-being of families and children, their rights and against those who might make decisions on their behalf and their costs. They conclude, that “much more information is needed about the social and behavioral consequences of the availability of personal biomarker information for children before evidence-based judgements can be made about the ethical issues raised by such technologies”.

In any case, most relevant ethical aspects should be kept in mind [29] and need to be discussed before the implementation of screening programs. Examples given are consent and respect of persons, stigma and participation rates, the cost-benefit analysis of a screening program, consequences of false-positive and false-negative test results, confidentiality and appropriate follow-up to positive screening results, as well as the use of screen results for criminal prosecution [30]. Also the right to not know [31] should be considered. This point is especially delicate in the case of minors where parents or other legal guardians make decisions on behalf of the child. In general, one could ask if biomarker research with the focus on a negative risk profile and its consequences is the right way to go. Or would it be even more fruitful to focus on biomarkers reflecting protective factors which make people more resilient towards mental diseases? We think both perspectives need to be respected within the framework of a good guidance by mental health specialists.

## Integrated research as the rule

The enthusiasm reflected in many recent papers on biomarkers in child mental health seems to be merely driven by the objective to be part of an “edge of science”, without critically discussing the pro and cons of such a research. This holds also true for the recent opinion of Pine and Leibenluft [32] who somewhat uncritically try to align psychiatry with biomarker research of other medical disciplines and suggest that “research on any biomarker that predict mental disorder course or outcome is valuable but priority should be given to biomarker research that includes a mechanistic focus”. An additional psycho-social perspective is missing.

Not only that studies on biomarkers need to deal with the pathophysiological background of the disorder/behavioral problem but they should also investigate the psychometric properties and disorder/dimensional specificity of the biomarker in question. Further, they should include in their design issues that influence the translational use of biomarkers in child psychiatry and beyond, i.e. sound scientific methods, as well as social, ethical, legal/regulatory and commercial aspects need to be considered. This is, for example, of utmost importance if the use of biomarkers might find its way to public health related practice. Here, risk profiling might serve for the prediction of behavior of minors and thus health insurance aspects (i.e. higher rates) as well as preventive interventions in very young children without obvious mental health problems (e.g. healthy siblings of ADHD patients) could be the focus with the risk of misuse. Therefore, we should deepen our mind that biomarkers should serve to improve the well-being of children and society in general [28] and we should take great care to avoid harm to children and their families; thus researchers should take a broader view than to focus on just one biological parameter or complex compositions of biomarkers. Moreover, integration of bio-psycho-social issues should be a mandatory part of research planning.

## References

[1] Kapur S, Phillips AG, Insel TR. Why has it taken so long for biological psychiatry to develop clinical tests and what to do about it? Mol Psychiatry. 2012;17:1174-9. 10.1038/mp.2012.105 22869033

[2] Adisetiyo V, Jensen JH, Tabesh A, Deardorff RL, Fieremans E, Di Martino A, Gray KM, Castellanos FX, Helpern JA (2014). Multimodal MR imaging of brain iron in attention deficit hyperactivity disorder: a noninvasive biomarker that responds to psychostimulant treatment?. Radiology.

[3] Campbell DJ, Chang J, Chawarska K (2014). Early generalized overgrowth in autism spectrum disorder: prevalence rates, gender effects, and clinical outcomes. J Am Acad Child Adolesc Psychiatry.

[4] Scassellati C, Bonvicini C, Faraone S, Gennarelli M (2012). Biomarkers and attention-deficit/hyperactivity disorder: a systematic review and meta-analyses. J Am Acad Child Adolesc Psychiatry.

[5] Rothenberger A (1982). Event-related potentials in children. Developments in neurology.

[6] Rothenberger A (1990). Brain and behavior in child psychiatry.

[7] Van Beveren NJM, Hoogendijk WJG (2011). Clinical utility of serum biomarkers for major psychiatric disorders. Int Rev Neurobiol.

[8] Peterson BS (2015). Editorial: Research Domain Criteria (RDoC): a new psychiatric nosology whose time has not yet come. J Child Psychol Psychiatry.

[9] Biomarkers Definitions Working Group (2001). Biomarkers and surrogate endpoints: preferred definitions and conceptual framework. Clin Pharmacol Ther.

[10] Thome J, Ehlis A-C, Fallgatter A (2012). Biomarkers for attention-deficit/hyperactivity disorder (ADHD). A consensus report of the WFSBP task force on biological markers and the World Federation of ADHD. World J Biol Psychiatry.

[11] O’Connor TG (2014). Editorial: updating biological bases of social behavior. J Child Psychol Psychiatry.

[12] Rommelse N, Zeeuw P (2014). Neurobiological measures to classify ADHD: a critical appraisal. Eur Child Adolesc Psychiatry.

[13] Wangler S, Gevensleben H, Albrecht B, Studer P, Rothenberger A (2011). Neurofeedback in children with ADHD: specific event-related potential findings of a randomized controlled trial. Clin Neurophysiol.

[14] Genius J, Klafki H, Benninghoff J, Esselmann H, Wiltfang J (2012). Current application of neurochemical biomarkers in the prediction and differential diagnosis of Alzheimer's disease and other neurodegenerative dementias. Eur Arch Psychiatry Clin Neurosci.

[15] Krüger-Brand HE (2015). Prognose-Tool für Psychosen. Dt Ärztebl..

[16] Fu CH, Costafreda SG (2013). Neuroimaging-based biomarkers in psychiatry: clinical opportunities of a paradigm shift. Can J Psychiatry.

[17] Mwangi B, Tian TS, Soares JC (2014). A review of feature reduction techniques in neuroimaging. Neuroinformatics.

[18] Bansal R, Hao X, Peterson BS (2015). Morphological covariance in anatomical MRI scans can identify discrete neural pathways in the brain and their disturbances in persons with neuropsychiatric disorders. Neuroimage.

[19] Bansal R, Hao X, Liu J, Peterson BS (2014). Using Copula distributions to support more accurate imaging-based diagnostic classifiers for neuropsychiatric disorders. Magn Reson Imaging.

[20] August JM, Rothenberger A, Baudewig J, Roessner V, Dechent P (2015). May functional imaging be helpful for behavioral assessment in children? Regions of motor and associative cortico-subcortical circuits con be differentiated by laterality and rostrality. Front Hum Neurosci.

[21] Faraone S (2014). Advances in the genetics of attention-deficit/hyperactivity disorder. Biol Psychiatry.

[22] Maughan B, Sonuga-Barke E (2014). Editorial: translational genetics of child psychopathology: a distant dream?. J Child Psychol Psychiatry.

[23] Hebebrand J, Antel J (2014). Squaring the circle? On the search for circulating biomarkers in polygenic psychiatric disorders. Eur Child Adoelsc Psychiatry.

[24] Albrecht B, Brandeis D, Uebel-von Sanderleben U, Valko L, Henrich H, Xu X, Drechsler R, Heise A, Kuntsi J, Müller UC, Asherson P, Steinhausen HC, Rothenberger A, Banaschewski T (2014). Genetics of preparation and response control in ADHD: the role of DRD4 and DAT1. J Child Psychol Psychiatry.

[25] Dolgin E (2014). FDA clearance paves way for computerized ADHD monitoring. Nat Med.

[26] Tomasik J, Schwarz E, Guest PC, Bahn S (2012). Blood test for schizophrenia. Eur Arch Psychiatry Clin Neurosci.

[27] Rutter M, Solantus T (2014). Translation gone awry: differences between commonsense and science. Eur Child Adolesc Psychiatry.

[28] Sing I, Rose N (2009). Biomarkers in psychiatry. Nature.

[29] Koelch M, Fegert JM (2010). Ethics in child and adolescent psychiatric care: an international perspective. Int Rev Psychiatry.

[30] Zizzo N, Di Pietro N, Green C, Reynolds J, Bell E, Racine E (2013). Comments and reflections on ethics in screening for biomarkers of prenatal alcohol exposure. Alcohol Clin Exp Res.

[31] Shaikh-Lesko R (2014) The right to not know. Scientist. http://www.the-scientist.com/?articles.view/article/No/39614/title/The-Right-to-Not-Know. 2014.

[32] Pine DS, Leibenluft E (2015). Biomarkers with a mechanistic focus. JAMA Psychiatry.

